# Quadriceps tendon autograft diameters are routinely above 8 mm, and preoperative size estimation before anterior cruciate ligament reconstruction may not be necessary for this graft type: A systematic review

**DOI:** 10.1002/ksa.12558

**Published:** 2024-12-17

**Authors:** Joshua Dworsky‐Fried, Amanda Hadwen, Luca Bernardini, Prushoth Vivekanantha, Alberto Grassi, Matthieu Ollivier, Darren de Sa

**Affiliations:** ^1^ Michael DeGroote School of Medicine McMaster University Hamilton Ontario Canada; ^2^ School of Graduate Studies, Faculty of Health Sciences McMaster University Hamilton Ontario Canada; ^3^ Division of Orthopaedic Surgery, Department of Surgery McMaster University Hamilton Ontario Canada; ^4^ IRCCS, Istituto Ortopedico Rizzoli Bologna Italy; ^5^ Institut du Mouvement et de l'appareil Locomoteur, Hôpital Sainte‐Marguerite Aix‐Marseille Université Marseille France

**Keywords:** ACL reconstruction, anthropometric, MRI, parameters

## Abstract

**Purpose:**

To identify values of reported quadriceps tendon (QT) autograft diameter and length in anterior cruciate ligament reconstruction (ACLR), and to identify methods to predict these parameters.

**Methods:**

A search was conducted across three databases from inception to 30 March 2024. Data on study characteristics, demographics, anthropometric data, imaging techniques, and QT autograft or harvested QT tendon parameters were extracted. Values quantifying the statistical strength of associations between imaging or anthropometric characteristics and graft or tendon parameters as well as for associations between these parameters and postoperative outcomes were recorded.

**Results:**

A total of 3633 patients were included. The weighted mean QT autograft diameter and length were 8.9 (standard deviation [SD]: 0.7, range: 7.8–10.4) mm and 8.1 (SD: 1.3, range: 5.6–9.3) cm, respectively. A total of 93.8% of studies that reported mean QT autograft diameter found a value of 8 mm or greater. The QT groups had similar or significantly greater mean autograft diameter compared to the hamstring tendon (HT) groups in 91.7% of studies that reported significance. Regarding MRI measurements, 71.4% of the correlation coefficients reported showed a moderate positive correlation, 28.6% showed a low positive correlation and 14.3% showed a high positive correlation. Regarding anthropometric characteristics, 33.3% and 16.7%, 16.7% and 14.3% of studies that reported on the relationship between QT autograft diameter and height, weight, body mass index or age, respectively, found a low positive statistically significant correlation. Only statistically nonsignificant associations were reported between QT autograft parameters and post‐operative outcomes and complications.

**Conclusions:**

QT autografts used in ACLR have a mean diameter of 8 mm or greater and are consistently larger than HT autografts. Preoperative MRI measurements are better than anthropometric characteristics at predicting QT autograft parameters; however, preoperative prediction may not be necessary for this graft type. QT autograft parameters were not found to be significantly associated with any post‐operative complication or outcome.

**Level of Evidence:**

Level IV.

**Abbreviations**
ACLR: anterior cruciate ligament reconstruction
BMI: body mass index
BPTB: bone‐patellar tendon‐bone
CI: confidence interval
CSA: cross‐sectional area
GT: gracilis tendon
HT: hamstring tendon
MRI: magnetic resonance imaging
PRISMA: Preferred Reporting Items for Systematic Reviews and Meta‐Analyses
PT: patellar tendon
QT: quadriceps tendon
R‐AMSTAR: Revised Assessment of Multiple Systematic Reviews
SD: standard deviation
ST: semitendinosus tendon
T: Tesla

## INTRODUCTION

Autografts that are used for anterior cruciate ligament reconstruction (ACLR) can vary significantly in size depending on autograft type and patient factors. Recent literature shows that post‐operative outcomes can be significantly affected depending on the size of the graft used. A recent meta‐analysis demonstrated that ACLR failure rates decreased with increasing graft diameter from 7 to 9 mm [36]. A previous cohort study using data from Scandinavian registries showed that revision surgery is 0.86 times less likely with every 0.5 mm increase in hamstring tendon (HT) autograft diameter from 7 to 10 mm [71]. In 2019, the same group published that an HT graft with diameter ≥9 mm had a reduced risk of early ACL revision surgery when compared to patellar tendon (PT) [72]. Graft length also informs post‐operative outcomes and hamstring autograft length is recommended to be a minimum of 6 cm [44].

Since graft size has such a large effect on post‐operative outcomes, predicting graft diameter preoperatively can inform surgical decisions and help ensure the optimal result for the patient [59]. Recently there have been multiple papers that describe methods to predict graft diameter [5, 7, 13, 26, 81]. A recent systematic review found that anthropometric characteristics and magnetic resonance imaging (MRI) parameters can help predict hamstring tendon graft diameter, although MRI parameters are superior in this. Despite this, height has been reported as the most reliable anthropometric characteristic that predicts graft diameter [3]. Some literature has also shown that anthropometric characteristics can be used to predict hamstring graft length, in addition to diameter [25]. A retrospective study from 2020 showed that for ACLR using hamstring autografts, cross‐sectional area (CSA) of the semitendinosus and gracilis tendons measured using preoperative MRI correlate with final autograft diameter [59].

Quadriceps tendon (QT) autografts are becoming increasingly popular as a graft source for ACLR. QT autografts are a valuable graft type which may provide advantages over more common autografts [15]. Because of the relatively recent increase in the frequency of QT autograft use in ACLR [21, 31, 70, 84], there is ongoing discourse surrounding the optimal graft parameters. For example, some studies suggest a possible correlation between arthrofibrosis and larger QT graft sizes [28]. A 2023 retrospective study demonstrated that there was an increase in the incidence of arthrofibrosis in all‐soft‐tissue QT grafts in female patients, in male patients with a femoral tunnel diameter of 9.25 mm or greater, and in patients with concomitant meniscal repair [28]. Baseline parameters for QT autografts would be beneficial for surgeons to plan their operations and to understand whether a QT autograft is a viable option for their patients.

Therefore, this review aims to provide three outcomes related to QT autograft parameters. First, the values of QT autograft diameters and length in patients undergoing ACLR; and comparing these values to those of HT and bone‐patellar tendon‐bone (BPTB) autografts. Second, to outline strategies currently used to predict QT autograft parameters. Third, to describe any relationships between QT autograft parameters and post‐operative outcomes. It is hypothesized that the average QT autograft diameter would be over 8 mm, and that preoperative MRI of QT CSA would be effective in predicting intraoperative graft size. Information from this review can be used clinically to guide decision‐making regarding using QT autografts in ACLR.

## METHODS

The PRISMA (Preferred Reporting Items for Systematic Reviews and Meta‐Analyses) and R‐AMSTAR (Revised Assessment of Multiple Systematic Reviews) guidelines for coordinating and reporting systematic reviews were followed during the development of this research [43, 49].

### Search criteria

Three online databases (EMBASE, PubMed and MEDLINE) were searched from database inception to 30 March 2024, to identify studies which either (1) outlined diameters or lengths of QT autografts prepared for ACLR or harvested QT, (2) reported on factors used to predict harvested tendon or graft diameter or length and post‐operative outcomes or (3) reported on correlations between previously harvested tendon or graft diameter and length and post‐operative outcomes. Comprehensive search terms including ‘quadriceps’, ‘anterior cruciate ligament’ or ‘ACL’, and ‘diameter’ or ‘length’ or ‘width’ or ‘size’ were used (Supporting Information S1: Table 1). Studies were selected for inclusion if they met the following criteria: (1) studies which used QT autografts for ACLR, (2) reported on either values of harvested QT or QT graft diameters or length, factors used to predict these parameters or (3) correlations between these metrics and post‐operative outcomes (4) human studies and (5) studies published in the English language. Exclusion criteria included (1) systematic reviews/meta‐analysis (2) textbook chapters, (3) conference abstracts, (4) editorial commentaries, (5) biomechanical or cadaveric/animal studies, or (6) technical studies. References of included studies and pertinent review papers were manually searched to ensure that all means of study identification were exhausted.

### Screening

Independent and blinded title and abstract screening were conducted by two authors (J. D. F. and A. H.), with conflicts resolved through consensus or consultation with a more senior author (P. V.). During the full‐text screening stage, studies were independently screened by the same two authors, and disagreements were resolved in a similar manner.

### Assessment of agreement

Inter‐reviewer agreement was evaluated using the *κ*‐statistic for screening. A priori classification was determined using the following criteria: *κ* of 0.91–0.99 was considered to be almost perfect agreement; *κ* of 0.71–0.90 was considered to be considerable agreement; *κ* of 0.61–0.70 was considered to be high agreement; κ of 0.41–0.60 was considered to be moderate agreement; *κ* of 0.21–0.40 was considered to be fair agreement; and a *κ* value of 0.20 or less was considered to be no agreement [45].

### Quality assessment

For randomized control trials (RCTs), the Detsky Quality Assessment Scale was used [17]. The scale contains 14 questions categorized as (1) randomization, (2) outcome measures, (3) inclusion and exclusion criteria and reasons for patient exclusion, (4) interventions, and (5) statistical analysis [17]. Equal weight was given to each category and each category could obtain a maximum of four points [17]. If there were negative findings, an additional question was added to the statistical analysis category (e.g., were confidence intervals [CIs] calculated or post‐hoc power calculations performed?) [17]. Maximum scores for trials with positive and negative findings were 20 and 21 points, respectively [17].

For non‐randomized studies, the Methodological Index for Non‐Randomized Studies (MINORS) criteria was used [69]. Based on the MINORS criteria, noncomparative and comparative studies could get a maximum score of 16 and 24, respectively [69]. For noncomparative studies, classification was a priori based on a previous systematic review: scores of 0–4 indicated very low‐quality evidence; 5–7 indicated low‐quality evidence; 8–12 indicated fair quality; ≥13 indicated high‐quality evidence [83]. Comparative studies were classified as follows: scores of 0–6 indicated very low‐quality evidence; 7–10 indicated low‐quality evidence; 11–15 indicated fair‐quality evidence; 16–20 indicated good quality evidence and ≥20 indicated high quality [83].

### Data extraction

Data were extracted in an electronic spreadsheet designed a priori (Google Sheets; Google LLC). Extracted data, when available, included study characteristics (e.g., author(s), year of publication and level of evidence) as well as demographic and anthropometric data, including patient age, sex, BMI, height, weight, thigh circumference, and thigh length. Graft characteristics such as, presence of a bone block, graft diameter and graft length were also extracted. For studies which included patients receiving non‐QT autografts, comparison of such graft characteristics with the QT patients was included. Additionally, the width and length of the harvested QT were recorded. For studies which included imaging, parameters such as imaging type and its description (e.g., magnification, number of teslas, type of cut) were extracted. For example, 2× versus 4× options for magnification, 1.5 T versus 3.0 T for Teslas, and axial versus sagittal versus coronal for types of cuts [82]. When ranges of measurements or minimum requirements were reported, they were not included in the analysis.

Associations between imaging parameters or anthropometric characteristics and QT graft or QT diameter/width or length were also extracted. Furthermore, associations between these parameters and post‐operative outcomes were recorded. Thus, the type of statistics used for each association (e.g., Pearson correlation coefficients, odds ratio) and its reported value were extracted. Correlation coefficients were interpreted using guidelines proposed by a previous study: values ranging from ±0.91 to 1.00 indicated very high positive/negative correlation, ±0.70 to 0.90 indicated high positive/negative correlation, ±0.50 to 0.70 indicated moderate positive/negative correlation, ±0.30 to 0.50 indicated low positive/negative correlation and 0.0–0.30 indicated negligible correlation [53]. Odds ratios were interpreted as such: values greater than 1 indicated that exposure was associated with higher outcome odds, values equal to 1 indicated that exposure did not affect the odds of the outcome, and values less than 1 indicated that exposure was associated with lower outcome odds [75]. Significance was also recorded, with *p* values less than 0.05 indicating significance.

## RESULTS

### Literature search

**Figure 1:**
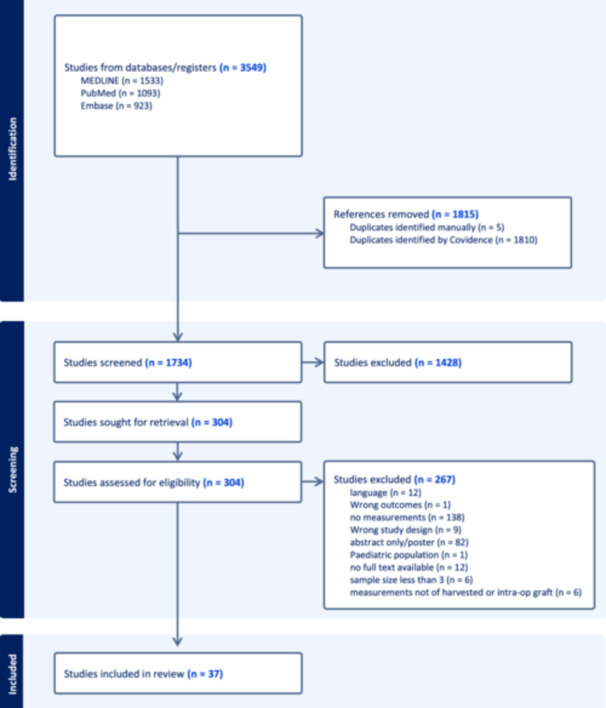
Preferred Reporting Items for Systematic Reviews and Meta‐analyses flow diagram representing a systematic review comparing QT autograft diameter and length in ACLR. ACLR, anterior cruciate ligament reconstruction; QT, quadriceps tendon.

A total of 3549 studies across EMBASE, PubMed and MEDLINE were identified. After removing 1815 duplicate studies, there were 1734 studies remaining for title and abstract screening. After applying the aforementioned eligibility criteria, 37 studies were identified and included [1, 6, 9, 10, 11, 12, 16, 19, 20, 24, 27, 28, 32, 35, 37, 38, 40, 42, 46, 48, 54, 57, 58, 60, 61, 62, 64, 65, 66, 74, 76, 77, 79, 80, 84, 86, 89]. There was an almost perfect agreement during the title and abstract screening stage (*κ* = 0.87, 95% CI = 0.84–0.90) and substantial agreement during the full‐text screening stage (κ = 0.74, 95% CI = 0.63–0.85) (Figure 1).

### Study quality

Overall, 4 (10.8%) [32, 42, 61, 79], 4 (10.8%) [1, 47, 57, 66], 27 (73.0%) [6, 9, 10, 11, 12, 19, 20, 27, 28, 35, 37, 38, 40, 48, 54, 58, 60, 62, 64, 65, 74, 76, 77, 80, 84, 86, 89] and 2 (5.4%) [16, 24] studies were identified as Level I, II, III and IV studies, respectively. The mean MINORS score across 35 studies was calculated to be 62.7% (standard deviation [SD]: 10.7%, range: 25%–75%) [1, 6, 9, 10, 11, 12, 16, 19, 20, 24, 27, 28, 35, 37, 38, 40, 42, 47, 48, 54, 57, 58, 60, 61, 62, 64, 65, 66, 74, 76, 77, 80, 84, 86, 89], and the Detsky scores from the two included studies were 90.9% and 81.8% (Table 1) [32, 79].

**Table 1:** Demographic and anthropometric characteristics.

First author (year of publication)	Journal	Level of evidence	Detsky/MINORS score (%)	Graft types: number of patients	Females (%)	Age (SD) [range] (years)	BMI (SD) [range] (kg/m^2^)	Height (SD) [range] (cm)	Weight (SD) [range] (kg)	Thigh circumference (SD) [range] (cm)	Thigh length (SD) [range] (cm)
Abouheif (2023)	International Orthopaedics	II	68.75	QT: 30	6.7	25.73 (2.41) [21–31]	24.98 (1.61) [22–27]	175.17 (8.12) [160–190]	NR	61.17 (6.65) [45–75]	NR
Baghdadi (2021)	American Orthopaedic Society for Sports Medicine	III	43.75	QT: 169	42	Median: 15 (NR) [9–17]	23.8 (4.9) [14.5–42.5]	168.7 (10.5) [133.9–198.9]	68.4 (18.2) [28.8–134.6]	NR	NR
Bram (2024)	The Journal of Arthroscopic and Related Surgery	III	66.67	QT: 40 HT: 44	QT: 37.5 HT: 65.9	QT: 15.8 (1.5) [NR] HT: 15.8 (1.6) [NR]	QT: 24.7 (5.6) [NR] HT: 23.3 (4.5) [NR]	NR	NR	NR	NR
Brinkman (2023)	The Journal of Arthroscopic and Related Surgery	III	70.83	QT: 37 HT: 46	QT: 54.1 HT: 52.1	QT: 23.4 (7.0) [NR] HT: 24.7 (7.1) [NR]	QT: 24.9 (3.4) [NR] HT: 24.9 (3.1) [NR]	NR	NR	NR	NR
Brinkman (2022)	American Orthopaedic Society for Sports Medicine	III	70.83	ASTQT: 32 BPTB: 26	ASTQT: 62.5 BPTB: 53.8	ASTQT: 18.3 (2.7) [NR] BPTB: 18.2 (2.8) [NR]	ASTQT: 24.3 (3.5) [NR] BPTB: 25.2 (3.1) [NR]	NR	NR	NR	NR
Cavaignac (2017)	American Orthopaedic Society for Sports Medicine	III	70.83	QT: 45 HT: 41	QT: 45 HT: 42	QT: 32.1 (8) [NR] HT: 30.9 (9) [NR]	QT: 22.4 (3.1) [NR] HT: 24.3 (2.9) [NR]	NR	NR	NR	NR
Daniel (2024)	The Journal of Arthroscopic and Related Surgery	IV	75	QT: 60	68	16.8 (NR) [13–23)	24.1 (NR) [22.8–25.4]	NR	NR	NR	NR
Eggeling (2021)	American Orthopaedic Society for Sports Medicine	III	70.83	QT: 43 HT: 46	QT: 37.2 HT: 41.3	QT: 32.9 (9.1) [25–44] HT: 27.6 (8.6) [18–41]	QT: 25.9 (4.5) [18–36] HT: 26.2 (5.2) [18–42]	NR	NR	NR	NR
Gagliardi (2022)	Skeletal Radiology	Not defined, 3b	56.25	QT: 102	54	15.9 (1.9) [NR]	23.6 (4.3) [NR]	168.7 (10.2) [NR]	66.8 (14.8) [NR]	NR	NR
Goto (2022)	Knee Surgery, Sports Traumatology, Arthroscopy	IV	70.83	QT: 73	100	33.8 (11.4) [NR]	22.7 (3.5) [NR]	165.5 (5.6) [NR]	62.2 (9.4) [NR]	NR	NR
Greif (2022)	The Journal of Arthroscopic and Related Surgery	III	66.67	TFSF: 62 TSF: 62	TFSF: 12.9 TSF: 32.3	TFSF: 27.74 (25.95–29.53) [NR] TSF: 31.21 (28.56–33.86) [NR]	TFSF: 26.78 (25.58–27.97) [NR] TSF: 27.78 (25.79–28.29) [NR]	NR	NR	NR	NR
Haley (2023)	The Journal of Arthroscopic and Related Surgery	III	56.25	QT: 721	46	No Arthrofibrosis—Male: 20.89 (6.94) No Arthrofibrosis—Female: 18.18 (4.83) Arthrofibrosis—Male: 22.35 (8.68) Arthrofibrosis—Female: 20.00 (5.22) 9–53	No Arthrofibrosis—Male: 24.60 (4.29) No Arthrofibrosis—Female: 22.48 (3.01) Arthrofibrosis—Male: 25.97 (5.16) Arthrofibrosis—Female: 22.46 (2.09)	NR	NR (NR) [30.4–147.4)	NR	NR
Horstmann (2022)	Archives of Orthopaedic and Trauma Surgery	Undefined, 1b	90.91	QT: 24 HT: 27	QT: 12.5 HT: 55.6	QT: 24.1 (3.6) [NR] HT: 32.7 (11.4) [NR]	QT: 24.9 (3.8) [NR] HT: 25.2 (4.0) [NR]	QT: 177.9 (7.3) [NR] HT: 171.2 (8.2) [NR]	HT: 73.6 (11.3) QT: 79.0 (13.6)	NR	NR
Iriuchishima (2017)	Knee Surgery, Sports Traumatology, Arthroscopy	III	68.75	QT: 20	90	49 (8) [NR]	NR	NR	NR	NR	NR
Johnston (2021)	Journal of Isakos	III	70.83	QT: 37 HT: 74	QT: 21.6 HT: 21.6	QT: 20.0 (NR) [15–34] HT: 20.5 (NR) [15–32]	NR	QT: 178.2 (7.6) [NR] HT: 178.4 (9.1) [NR]	QT: 78.8 (11.3) [NR] HT: 78.6 (11.9) [NR]	NR	NR
Johnston (2022)	Knee Surgery, Sports Traumatology, Arthroscopy	III	75	QT: 35 HT: 70	21.9	QT: 20 (NR) [15–34] HT: 20 (NR) [15–32]	NR	QT: 178.5 (7.8) [NR] HT: 178.1 (8.9) [NR]	QT: 79.3 (11.3) [NR] HT: 78.0 (11.4) [NR]	NR	NR
Josipovic (2023)	Acta Clinica Croatica	Undefined, 3b	62.5	QT: 9	44.4	27 (9.5) [NR]	NR	NR	NR	NR	NR
Kohl (2014)	The Knee	Undefined, 1b	75	QT: 15	20	12.8 (2.6) [6.2–15.8]	NR	NR	NR	NR	NR
Lee (2024)	Clinics in Orthopaedic Surgery	Undefined, 2b	56.25	Full thickness QTPB: 38 Partial‐thickness QTPB: 40	Full thickness QTPB: 13.1 Partial thickness QTPB: 12.5	Full thickness: 32.1 (11.5) [NR] Partial thickness: 31.4 (10.0) [NR]	Full thickness: 25.0 (4.3) [NR] Partial thickness: 25.2 (3.8) [NR]	NR	NR	NR	NR
Letter (2023)	American Orthopaedic Society for Sports Medicine	III	66.67	Full thickness QT: 12 Partial thickness QT: 14	Full thickness: 33 Partial thickness: 7.1	Full thickness: 28.5 (6.72) [NR] Partial thickness: 26.4 (4.56) [NR]	NR	Full thickness: 176 (9) [NR] Partial thickness: 175 (7) [NR]	Full thickness: 89.5 (25.7) [NR] Partial thickness: 84.1 (14.8) [NR]	NR	NR
Mutsuzaki (2019)	Journal of Orthopaedics	III	56.25	QT: 8	75	41.6 (10.6) [NR]	NR	157.8 (7.7) [NR]	55.8 (15.7) [NR]	NR	NR
Panos (2021)	American Orthopaedic Society for Sports Medicine	II	62.5	QT: 23 HT: 55	QT: 30.4 HT: 47.2	QT: 22.0 (6.1) [14.4–35.2] HT: 22.2 (5.5) [14.2–38.2]	QT: 24.5 (3.4) [19.6–32.6] HT: 24.6 (3.0) [18.4–34.0]	NR	NR	NR	NR
Pennock (2019)	American Orthopaedic Society for Sports Medicine	III	66.67	QT: 27 HT: 56	QT: 32 HT: 31	QT: 14.8 (1.3) [NR] HT: 14.8 (1.4) [NR]	QT: 21.5 (3.2) [NR] HT: 23.9 (5.7) [NR]	NR	QT: 61.3 (11.3) [NR] HT: 67.9 (18.0) [NR]	NR	NR
Pfeiffer (2023)	American Orthopaedic Society for Sports Medicine	III	54.17	QT: 10 ST: 10	QT: 60 ST: 80	QT: 27 (10.1) [NR] ST: 31.7 (12.5) [NR]	NR	NR	NR	NR	NR
Pomenta Bastidas (2022)	Acta Orthopaedica Belgica	Undefined, 1b		QT: 20 HT: 24	QT: 15 HT: 33.3	QT: 30.2 (2.38) [NR] HT: 31.1 (2.60) [NR]	QT: 24.12 (1.4) [NR] HT: 24.8 (1.2) [NR]	NR	NR	NR	NR
Renfree (2023)	American Orthopaedic Society for Sports Medicine	III	70.83	QT: 32 BPTB: 36	QT: 59.4 BPTB: 52.8	QT: 17.6 (2.8) [NR] BPTB: 18.3 (2.5) [NR]	QT: 23.6 (NR) [18–31] BPTB: 24.5 (NR) [21–30]	NR	NR	NR	NR
Sadoghi (2023)	Archives of Orthopaedic and Trauma Surgery	III	43.75	QT: 102	49	34.9 (18.9) [NR]	24 (4.4) [NR]	171.4 (0.1) [NR]	71.4 (17) [NR]	NR	NR
Schmücker (2021)	American Orthopaedic Society for Sports Medicine	III	58.33	QT: 223 HT: 252	39.2	QT: 26.6 (8.6) [NR] HT: 27.4 (8.9) [NR]	NR	NR	NR	NR	NR
Schulz (2013)	Open Access Journal of Sports Medicine	II	68.75	QT: 55	43.6	31.7 (NR) [15–58]	23.5 (NR) [17.9–35.8]	NR	NR	NR	NR
Supreeth (2023)	Musculoskeletal Surgery	III	62.5	QT: 19 BPTB: 31 STG: 34	0	29 (8.4) [NR]	NR	NR	NR	NR	NR
Takeuchi (2021)	Knee Surgery, Sports Traumatology, Arthroscopy	III	56.25	QT: 31	32.3	20.9 (5) [NR]	26.2 (5) [NR]	177.1 (10.3) [NR]	81.4 (16.6) [NR]	NR	NR
Takeuchi (2022)	Knee Surgery, Sports Traumatology, Arthroscopy	III	62.5	QT: 30	40	19.9 (5) [NR]	24.1 (3.4) [NR]	175.4 (11.5) [NR]	74.5 (14) [NR]	NR	NR
Tang (2024)	European Journal of Orthopaedic Surgery and Traumatology	I	81.82	QT: 17 HT: 16	9.1	QT: 28.06 (6.24) [NR] HT: 28.21 (8.55) [NR]	NR	QT: 175.65 (5.95) [NR] HT: 173.44 (7.58) [NR]	QT: 77.35 (13.54) [NR] HT: 77.56 (16.93) [NR]	QT (preoperative): 49.32 (5.1) HT (preoperative): 50.0 (5.8) QT (post‐operative): 48.71 (5.14) HT (post‐operative): 49.19 (5.46)	NR
Todor (2019)	Acta Orthopaedica et Traumatologica Turcica	III	62.5	QT: 39 HT: 33	31.9	QT: 30.64 (8.71) [18–53] HT: 28.60 (6.74) [18–46]	QT: 25.17 (4.38) [18.52–37.58] HT: 25.21 (2.92) [18.34–30.93]	NR	NR	NR	NR
Winkler (2022)	Knee Surgery, Sports Traumatology, Arthroscopy	III	50	QT: 106 BPTB: 88 HT: 8 Allograft: 55 Hamstring cont.: 1 BPTB cont.: 2	45	26.2 (9.4) [13–58]	26.9 (5) [19–48]	NR	NR	NR	NR
Yamasaki (2021)	Knee Surgery, Sports Traumatology, Arthroscopy	III	25	QT: 30	30	24.5 (2.6) [15–49]	NR	169.3 (2.1) [153–192]	NR	NR	NR
Zakko (2017)	Knee Surgery, Sports Traumatology, Arthroscopy	III	58.33	QT: 29 HT: 14 BPTB: 3 Allograft: 14 Hybrid: 2	37.1	25.1 (10.4) [13.4–50.2]	25.2 (5) [17.2–44.5]	152.4 (9.9) [152.4‐196.0]	77.1 (20.1) [45.5–140.6]	NR	NR

Abbreviations: ASTQT, all‐soft tissue quadriceps tendon; BPTB, bone‐patellar tendon‐bone; HT, hamstring tendon; QT, quadriceps tendon; QTPB, quadriceps tendon‐patellar bone, STG, semitendinosus tendon‐gracilis; TFSF, tibial‐femoral suspensory fixation.

### Study characteristics

The 37 included studies comprised 3633 patients. The weighted mean age of included patients was 24.4 (range of means: 12.8–49.0) years in 37 studies that reported values [1, 6, 9, 10, 11, 12, 16, 19, 20, 24, 27, 28, 32, 35, 37, 38, 40, 42, 46, 48, 54, 57, 58, 60, 61, 62, 64, 65, 66, 74, 76, 77, 79, 80, 84, 86, 89]. Females comprised 40.9% (range: 0%–100%) of all patients in 37 studies that reported values [1, 6, 9, 10, 11, 12, 16, 19, 20, 24, 27, 28, 32, 35, 37, 38, 40, 42, 46, 48, 54, 57, 58, 60, 61, 62, 64, 65, 66, 74, 76, 77, 79, 80, 84, 86, 89]. The weighted mean height of included patients was 171.1 (range of means: 157.8–178.5) cm in 16 studies that reported values [1, 6, 20, 24, 28, 32, 37, 38, 48, 54, 64, 76, 77, 79, 86, 89]. The weighted mean weight of included patients was 71.3 (range of means: 55.8–89.5) kg in 14 studies that reported values [6, 20, 24, 32, 37, 38, 48, 54, 58, 64, 76, 77, 79, 89]. The weighted mean BMI of patients was 24.5 kg/m^2^ (range of means: 21.5–27.8) kg/m^2^) in 25 studies that reported values (Table 1) [1, 6, 9, 10, 11, 12, 16, 19, 20, 24, 27, 28, 32, 46, 57, 58, 61, 62, 64, 66, 76, 77, 80, 84, 89].

### Graft parameters

The weighted mean QT autograft diameter, reported in 32 studies, was 8.9 (SD: 0.7, range: 7.8–10.4) mm [1, 6, 9, 10, 11, 12, 16, 19, 20, 24, 28, 32, 35, 37, 38, 40, 42, 48, 54, 57, 58, 60, 61, 62, 65, 66, 74, 76, 77, 79, 80, 84]. The mean QT autograft length was 8.1 (SD: 1.3, range 5.6–9.3) cm, reported in seven studies [1, 24, 27, 40, 54, 66, 80]. A total of 30 studies reported a mean QT autograft diameter of 8 mm or greater across all included patients (Table 2) [1, 6, 9, 10, 11, 12, 16, 19, 20, 24, 28, 32, 35, 37, 38, 40, 42, 48, 54, 57, 58, 60, 61, 62, 74, 76, 77, 79, 80, 84].

**Table 2:** Graft characteristics.

First author (year of publication)	Bone block used (yes/no/both)	Graft diameter (SD) [range] (mm)	Graft length (SD) [range] (cm)	QT graft diameter versus other autograft options (SD) [range] (mm)	QT graft length with other autograft options	Harvested QT width mean (mm)	Harvested QT length mean (cm)
Abouheif (2023)	No	QT: 8.20 (0.81) [7–10]	9.30 (1.51) [7–12]	NR	NR	NR	NR
Baghdadi (2021)	No	QT: 8.4 (0.7) [7–11]	NR (mean), 94% had graft length > 6.5 cm	NR	NR	10	NR
Bram (2024)	No	QT: 8.3 (0.7) [NR] HT: 8.7 (0.7) [NR]	NR	QT: 8.3 (0.7) [NR] HT: 8.7 (0.7) [NR] *p* = 0.01	NR	NR	NR
Brinkman (2023)	No	QT: 9.6 (6.1) [NR] HT: 7.9 (0.89) [NR]	NR	QT: 9.6 (6.1) [NR] HT: 7.9 (0.89) [NR] *p* < 0.001	NR	10	9
Brinkman (2022)	No	ASTQT: 9.8 (0.7) [NR] BPTB: 9.6 (0.6) [NR]	NR	ASTQT: 9.8 (0.7) [NR] BPTB: 9.6 (0.6) [NR] *p* = 0.392	NR	NR	NR
Cavaignac (2017)	Yes	QT: 9 (0.62) [NR] HT: 7.8 (0.79) [NR]	NR	QT: 9 (0.62) [NR] HT: 7.8 (0.79) [NR] *p* = NR	NR	NR	NR
Daniel (2024)	No	QT: 10.4 (NR) [10.2–10.5]	NR	NR	NR	NR	NR
Eggeling (2021)	Yes	QT: 8.2 (0.6) [7–9] HT: 8.5 (0.9) [7–10]	NR	QT: 8.2 (0.6) [7–9] HT: 8.5 (0.9) [7–10] *p* = 0.377	NR	NR	14
Gagliardi (2022)	Yes	Bone‐end: 10.6 (0.3) [NR] Soft‐tissue end: 9.6 (0.8) [NR]	NR	NR	NR	10	6.5
Goto (2022)	Yes	QT: 8.0 (0.5) [NR]	7.9 (0.4)	NR	NR	NR	NR
Greif (2022)	NR	NR	TFSF: 6.96 (6.90–7.01) [NR] TSF: 7.93 (7.72–8.13) [NR]	NR	TFSF: 6.96 (6.90–7.01) [NR] TSF: 7.93 (7.72–8.13) [NR] *p* < 0.00001	10	NR
Haley (2023)	No	No Arthrofibrosis—Male: 9.01 (0.67) [NR] No Arthrofibrosis—Female: 8.93 (0.64) [NR] Arthrofibrosis—Male: 9.29 (0.62) [NR] Arthrofibrosis—Female: 9.01 (0.67) [NR]	NR	NR	NR	NR	NR
Horstmann (2022)	Yes	QT: 8.9 (0.5) [NR] HT: 7.9 (0.6) [NR]	NR	QT: 8.9 (0.5) [NR] HT: 7.9 (0.6) [NR] *p* = 0.001	NR	NR	NR
Iriuchishima (2017)	No	QT: 8.1 (1.4) [NR]	NR	NR	NR	5	8
Johnston (2021)	No	Proximal HT: 8.0 (NR) [7–9] Distal HT: 8.5 (NR) [7.5‐10] Proximal QT: 8.0 (NR) [7–10] Distal QT: 9.0 (NR) [7–10]	NR	Proximal HT: 8.0 (NR) [7–9] Distal HT: 8.5 (NR) [7.5–10] Proximal QT: 8.0 (NR) [7–10] Distal QT: 9.0 (NR) [7–10] *p* = NR	NR	12	NR
Johnston (2022)	No	QT: 8.5 (1) [7–10] HT: 8.0 (1) [7–9]	NR	QT: 8.5 (1) [7–10] HT: 8.0 (1) [7–9] *p* = 0.03	NR	12	NR
Josipovic (2023)	Both	9 (1) [NR]	9 (1) [NR]	NR	NR	10	NR
Kohl (2014)	No	8.2 (0.77) [NR]	NR	NR	NR	NR	8
Lee (2024)	Yes	NR	NR	NR	NR	10	6
Letter (2023)	No	Partial thickness QT: 9.43 (0.62) [NR] Full thickness QT: 9.58 (0.42) [NR]	NR	NR	NR	10	NR
Mutsuzaki (2019)	Yes	Femoral side: 8.7 (0.5) [NR] Tibial side: 9.5 (0.5) [NR]	5.59 (0.32) [NR]	NR	NR	NR	NR
Panos (2021)	No	Proximal QT: 8.17 (0.86) [NR] Distal QT: 8.76 (0.78) [NR] Proximal HT: 7.83 (0.56) [NR] Distal HT: 8.61 (0.72) [NR]	NR	Proximal QT vs. HT: *p* = 0.09 Distal QT vs. HT: *p* = 0.43	NR	12	NR
Pennock (2019)	No	QT: 9.6 (0.6) [NR] HT: 7.8 (0.7) [NR]	NR	QT: 9.6 (0.6) [NR] HT: 7.8 (0.7) [NR] *p* < 0.001	NR	NR	NR
Pfeiffer (2023)	No	QT: 8.7 (0.6) [NR] ST: 8.3 (0.9) [NR]	NR	QT: 8.7 (0.6) [NR] ST: 8.3 (0.9) [NR] *p* = NR	NR	NR	NR
Pomenta Bastidas (2022)	Yes	HT and QT: 8.39 (0.27) [NR]	NR	NR	NR	NR	8
Renfree (2023)	No	QT: 9.5 (NR) [8–11] BPTB: 9.7 (NR) [9–11]	NR	QT: 9.5 (NR) [8–11] BPTB: 9.7 (NR) [9–11] *p* = 0.50	NR	NR	NR
Sadoghi (2023)	No	NR	NR	NR	NR	NR	NR
Schmücker (2021)	No	QT: 7.9 (0.8) [NR] HT: 7.7 (0.8) [NR] *p* = 0.015	NR	QT: 7.9 (0.8) [NR] HT: 7.7 (0.8) [NR] *p* = 0.015	NR	NR	NR
Schulz (2013)	No	7.8 (NR) [6–9]	8.8 (NR) [7.5–10]	NR	NR	NR	NR
Supreeth (2023)	No	QT: 9.6 (0.8) [NR] BPTB: 9.8 (0.7) [NR] STG: 8.4 (0.8) [NR]	NR	QT: 9.6 (0.8) [NR] BPTB: 9.8 (0.7) [NR] STG: 8.4 (0.8) [NR]	NR	NR	NR
Takeuchi (2021)	No	10 (1)	NR	NR	NR	10	NR
Takeuchi (2022)	Both	9.9 (0.6)	NR	NR	NR	10	NR
Tang (2024)	No	QT: 9.74 (1.55) HT: 7.91 (0.2)	NR	QT: 9.74 (1.55) HT: 7.91 (0.2) *p* < 0.001	NR	NR	NR
Todor (2019)	No	QT femoral: 8.57 (0.56) [7.5–10] QT tibial: 9.03 (0.63) [8–11] HT femoral: 7.65 (0.6) [7–9] HT tibial: 8.04 (0.51) [7–9]	QT: 8.97 (0.58) [7.5–10]	NR	NR	NR	NR
Winkler (2022)	No	9.6 (0.7) [7.5–12]	NR	NR	NR	NR	NR
Yamasaki (2021)	No	NR	NR	NR	NR	NR	NR
Zakko (2017)	Both	NR	NR	NR	NR	NR	NR

Abbreviations: ASTQT, all‐soft tissue quadriceps tendon; BPTB, bone‐patellar tendon‐bone; HT, hamstring tendon; QT, quadriceps tendon; STG, semitendinosus tendon‐gracilis; TFSF, tibial‐femoral suspensory fixation.

Twelve studies directly compared mean diameters between QT and HT autograft groups, with mean values of 8.4 (SD: 0.6, range: 7.9–9.7) mm and 8.0 (SD: 0.3, range: 7.7–8.7) mm, respectively [9, 10, 19, 32, 37, 38, 57, 58, 60, 61, 65, 79]. Out of these 12 studies, 6 studies reported significantly greater autograft diameters in the QT group [10, 32, 38, 58, 65, 79], 1 study reported a significantly greater diameter in the HT group [9], and 5 studies reported no statistical difference between the two groups [19, 37, 57, 60, 61]. Two studies directly compared mean diameters between QT and BPTB autograft groups, with mean values of 9.7 mm in both groups [11, 62]. No significant differences were found between the two groups in either study (Table 2).

The QT autograft was harvested with a bone block in 10 studies [12, 19, 20, 24, 32, 40, 46, 54, 61, 77]; the weighted mean QT autograft diameter in these studies was 8.6 (SD: 1.4, range: 5–10.1) mm, compared to a weighted mean QT autograft diameter of 8.8 (SD: 0.7, range: 7.8–10.4) mm when harvested without a bone block, reported in 23 studies [1, 6, 9, 10, 11, 16, 28, 35, 37, 38, 42, 48, 57, 58, 60, 62, 65, 66, 74, 76, 79, 80, 84] (Table 2).

Thirteen studies reported harvested QT width with a mean value of 10.1 (SD: 1.8, range: 5‐12) mm [6, 10, 20, 27, 35, 37, 38, 40, 46, 48, 57, 76, 77] and seven studies reported harvested QT length with a mean value of 8.5 (SD: 2.6, range: 6–14) cm [10, 19, 20, 35, 42, 46, 61] (Table 2).

### Associations between predictive variables and QT autograft parameters

#### MRI

The studies which used MRI to predict QT autograft parameters [1, 6, 11, 20, 46, 54, 57, 64, 65, 66, 74, 76, 77, 86, 89] used either T1‐weighted, T2‐weighted or proton‐density weighted sequences. The majority of studies used a strength of 1.5 T, and when reported, most used sagittal cuts. Full details of imaging parameters are described in Table 3.

**Table 3:** Imaging and anthropometric characteristics.

	Imaging				Anthropometric	
First author (year of publication)	Imaging type	Description of Imaging (sequence, teslas, cut type)	Type of statistics	Statistical association	Type of statistics	Statistical association
Baghdadi (2021)	MRI	Sequence: T1 or proton density‐weighted Teslas: NR Cut type: Sagittal	Interclass correlation coefficient (ICC) analysis	Multivariate logistic regression model, quadriceps tendon (QT) thickness on sagittal images (*p* = 0.003) was significant predictor of a final graft diameter >8 mm. QT sagittal thickness >6.7 mm on MRI was 97.4% sensitive and 46.6% specific in predicting final graft diameter >8 mm.	Stepwise linear regression model, multivariate logistic regression model.	Height significantly correlated with graft length (*p* < 0.001) in stepwise linear regression model. In multivariate logistic regression model, age was significant predictor of final graft diameter >8 mm *p* = 0.04, weight: *p* = 0.33, height *p* = 0.37, BMI *p* = 0.44)
Gagliardi (2022)	MRI	Sequence: T2 fatty acid weighted Teslas: NR Cut type: NR	Pearson correlation coefficients, Spearman correlation	(1) Correlation between bone‐end graft diameter and MRI AP measure of quadriceps at 10 mm above patella: *p* = 0.76, *r* = −0.03, MRI diameter significantly underestimated bone‐end graft diameter (7.3 [1.1] compared to 10.6 [0.3], *p* < 0.001). (2) Correlation between soft‐tissue side graft diameter and MRI‐predicted graft diameter at most proximal available point above patella: *p* < 0.001, *r* = 0.51, MRI measurement significantly underestimated the harvested soft tissue graft diameter (7.4 [1.1] compared to 9.6 [0.8], *p* < 0.001) (3) On bone end, difference between harvested graft diameter and MRI measurement was 3.3 (1.2) mm (4) On soft‐tissue end, difference between harvested graft diameter and MRI measurement was 2.2 (1.0) mm	Multivariate regression model	Association of anthropometric features with tibial side graft diameter: Sex: *B*‐coefficient: 0.097 *p* value: 0.64 Age: *B*‐coefficient: 0.358 *p* value: 0.51 Height: *B*‐coefficient: 0.008 *p* value: 0.85 Weight: *B*‐coefficient: 0.019 *p* value: 0.77 BMI: *B*‐coefficient: −0.047 *p* value: 0.77 Skeletally mature: *B*‐coefficient: −0.708 *p* value: 0.008 + 7
Mutsuzaki (2019)	MRI, CT	Sequence: Proton density‐weighted Teslas: 3.0 T Cut type: sagittal, coronal, axial	CT: The increase in tunnel CSA was calculated as: CSA increase rate (%) = (CSA at 1 year or 2 years—CSA at 1 week) × 100/CSA at 1 week	Graft intensity on MRI: low, iso, high (*n*): 1 year—3, 4, 0 (*n* = 7); 2 years—3, 2, 0 (*n* = 5) The intensity of the graft was iso or low in the MRI evaluation. None of the cases had high intensity. The increased CSA rate in the tibial bone tunnel was not significantly different between 1 and 2 years post‐operatively	NR	NR
Sadoghi (2023)	MRI	Sequence: T2 weighted Teslas: 3.0 T Cut type: sagittal, axial	NR	NR	Spearman	Heightened not significantly correlated with QT thickness (*p* = NS), also NS for weight, BMI, sex, age
Takeuchi (2021)	MRI	Sequence: T2 weighted Teslas: 1.5 T Cut type: sagittal, axial	Pearson's correlation coefficient, linear regression analysis	Significant correlations between the intraoperative diameter and the thickness, CSA, and adjusted CSA as follows (fig. 4a–c): thickness, *R* = 0.434 (95% CI, 0.095–0.684), *p* = 0.015; CSA, *R* = 0.607 (95% CI, 0.322–0.791), *p* < 0.001; adjusted CSA, *R* = 0.540 (95% CI, 0.229–0.751), *p* = 0.002	Linear regression analysis	No significant relationship between anthropometric characteristics and intraoperative diameter
Takeuchi (2022)	MRI	Sequence: T2 weighted Teslas: 1.5 T Cut type: sagittal	Pearson's correlation coefficient, linear regression analysis	(1) MRI thickness was significantly larger than US thickness at 15 mm (*p* < 0.001) (2) US: significant correlation between the intraoperative diameter of the QT autograft and the US thickness at 15 and 30 mm, and US CSA at 30 mm as follows (fig. 6a–c): US thickness at 15 mm, *R* = 0.791 (95% CI, 0.603–0.896), *p* < 0.001; US thickness at 30 mm, *R* = 0.772 (95% CI, 0.570–0.886), *p* < 0.001; US CSA at 30 mm, *R* = 0.738 (95% CI, 0.515–0.868), *p* < 0.001 (3) MRI: significant correlation between the intraoperative diameter of the QT autograft and the MRI thickness at 15 mm, MRI CSA at 15 mm, and MRI adjusted CSA at 15 mm as follows (fig. 7a–c): MRI thickness at 15 mm, *R* = 0.449 (95% CI, 0.106–0.697), *p* = 0.013; MRI CSA at 15 mm, *R* = 0.531 (95% CI, 0.212–0.748), *p* = 0.003; MRI adjusted CSA at 15 mm, *R* = 0.543 (95% CI, 0.227–0.756), *p* = 0.002	Linear regression analysis	Height significantly correlated with the intraoperative diameter of QT autograft (*R* = 0.377) (*P* = 0.040), other demographics were not significant (no correlation coefficients given)
Winkler (2022)	NR	NR	NR	NR	Descriptive statistics, chi‐square (categorical), *t* test/Mann–Whitney *U* test (continuous)	*p* < 0.05 for graft diameter between age groups: <20: 9.7 ± 0.6 (8.0–11.0) 20–30: 9.6 ± 0.7 (8.0–12.0) >30: 9.4 ± 0.7 (7.5–11.0) *no correlation provided
Yamasaki (2021)	MRI	Sequence: proton density‐weighted Teslas: 3.0 T Cut type: sagittal	Pearson's correlation coefficient	*r *= 0.67, *p* < 0.001 significant positive correlation between QT measurements on MRI and under direct visualization *measured on MRI as described in U38 but showed strong positive correlation between MRI measurement and visual measurement (*r* = 0.85, *p* < 0.001)	*T*‐test, chi‐square (for sex)	Sex, age and height were not significantly related to the presence of an adequate QT length (*p* = n.s) There was a significant positive correlation between patient height and QT length (*r* = 0.67, *p* < 0.001)
Zakko (2017)	MRI	Sequence: T1 and T2 weighted Teslas: 1.5 T Cut type: sagittal, axial	Descriptive statistics	NR	Pearson, point‐biserial (for sex)	QT thickness and height (*r* = 0.3, *p* = 0.03) QT thickness and weight (*r* = 0.3, *p* = 0.01) QT thickness and BMI (*r* = 0.3, *p* = 0.04) Age: NS

Abbreviations: AP, anterior‐posterior; BMI, body mass index; CI, confidence interval; CSA, cross‐sectional area; CT, computed tomography; MRI, magnetic resonance imaging; US, ultrasound.

Three studies [46, 76, 77] comprising 139 patients reported a weighted mean QT CSA measured on MRI of 79.8 mm^2^ when QT grafts were full‐thickness, and one study reported a CSA of 50 mm^2^ when the QT autografts used were partial‐thickness [46]. Two studies comprising 61 patients found significant correlations between QT thickness, CSA and adjusted CSA, and intraoperative QT autograft diameter [76, 77]. Six correlation coefficients amongst these two studies were reported and all were found to be statistically significant. QT thickness, CSA and adjusted CSA were significantly correlated with QT intraoperative diameter in both studies. Values of statistical correlation and significance are found in Table 3.

One study reported that QT thickness on sagittal MRI images was a significant predictor of a final QT autograft diameter of ≥8 mm [6] and another found a significant correlation between soft‐tissue graft diameter and MRI anterior‐posterior (AP) measurement [20]. One study found a strong and significant correlation between QT lengths when measured on MRI sagittal images compared to under direct visualization [86]. Full details on imaging techniques and correlations between imaging and graft diameter are reported in Table 3.

#### Anthropometric parameters

Seven studies comprising 493 patients reported on the association between height and graft parameters [6, 20, 64, 76, 77, 86, 89]. Two studies reported a significant correlation between height and autograft diameter [48, 54]. Four studies reported no significant correlation between height and graft diameter [6, 20, 64, 76]. One study found a significant positive correlation between height and QT graft length [86], and also determined that a QT with a length >60 mm on both MRI and under direct visualization was significantly associated with an adequate graft length. Full details on statistical significance and correlations between anthropometric features and graft parameters are reported in Table 3.

Six studies comprising 463 patients reported on the correlation between weight and graft diameter [6, 20, 64, 76, 77, 89]. One study reported a significant correlation [89], whereas five studies reported no significant correlation with graft diameter (Table 3) [6, 20, 64, 76, 77].

Six studies comprising 463 patients reported on the correlation between BMI and graft diameter [6, 20, 64, 76, 77, 89]. One study reported a significant correlation [89] whereas five studies reported no significant correlation with graft diameter (Table 3) [6, 20, 64, 76, 77].

Seven studies comprising 493 patients reported on the correlation between age and graft diameter [6, 20, 64, 76, 77, 86, 89]. One study reported that age is a significant predictor of final graft diameter >8 mm [6]. Six studies reported no significant correlation with graft diameter (Table 3) [20, 64, 76, 77, 86, 89].

#### Associations between QT autograft parameters and post‐operative outcomes

A total of three studies reported a statistical association between QT autograft parameters and post‐operative outcomes and/or complications [28, 58, 60]. In these studies, it was shown that the correlations between QT autograft parameters in ACLR and post‐operative outcomes including rate of ACL retears, risk of developing arthrofibrosis and post‐operative vancomycin concentration in synovial fluid were all statistically non‐significant (Supporting Information S1: Table 2).

## DISCUSSION

The primary finding of this paper was that the mean diameter of QT autografts that are used in ACLR was large, with a value of 8.9 (SD: 0.7, range: 7.8–10.4) mm. Notably, 93.8% of studies that reported a mean QT autograft diameter recorded a measurement of 8 mm or greater. Furthermore, six out of twelve studies that reported a direct comparison found that QTs had a statistically significant greater mean autograft diameter compared to HTs.

One previous systematic review reported mean diameters of 7.9 mm and 8.3 mm for patients undergoing ACLR with either two semitendinosus tendon (ST) and gracilis tendon (GT) strands each or four ST strands, respectively [82], and another study reported a mean ST length of 2.5 cm [87]. Structurally, the QT is much larger than the HT and PT; QTs have almost two times the CSA when compared to HTs and PTs [4, 67]. In addition, the QT has a unique thickness, with values of approximately 18 mm in males and 16 mm in females, compared to a mean thickness of 7.7 mm found in quadrupled hamstring grafts [25, 51]. Thus, a full‐thickness QT autograft can be harvested while preserving a significantly greater portion of native tissue [85]. The increased size of the QT allows it to be inherently robust; QTs have a higher strain at failure and Young's modulus of elasticity compared to PTs and HTs and the ultimate load to tendon failure was found to be 1.36 times greater with QTs than with PTs [30, 67, 68, 73]. Additionally, a thin fatty layer exists between the superficial rectus femoris and deep vastus intermedius portions of the QT, which produces a plane to facilitate double‐bundle ACLR if desired [51]. QTs can be harvested with or without a bone block, and variation exists in fixation methods [18, 51]. Owing to these properties, QT autografts grant versatility and flexibility to surgeons performing ACLR [51]. There are various circumstances in which the use of a QT autograft in ACLR may be preferable to other autograft choices. For example, QT use may be indicated when preserving hamstring anatomy is preferred or harvesting the HT is not a viable option such as in revision ACLR surgery, with shorter individuals and females with inadequate tendon size, or in cases with significant hamstring trauma [78]. One study postulated that a recent large increase in QT autograft usage in revision ACLR may be due to emerging evidence of relatively higher rates of HT autograft failures [84]. QT use in ACLR has been demonstrated to result in equivalent or even better post‐operative stability and functional and clinical outcomes when compared to other autograft types [12, 22, 23, 29, 34, 47, 50, 52, 70, 78]. Moreover, QT autografts have been shown to result in less donor site morbidity [23, 63, 70], post‐operative anterior knee pain, and numbness compared to both HT and BPTB [2, 12, 22, 34, 39, 52]. Therefore, it seems that the QT autograft is a viable alternative to other autograft types in ACLR and may even result in superior outcomes in certain circumstances.

Despite reports that variability exists in the length of available graft tissue due to the morphology of the QT, a QT autograft of consistent length can be harvested without disruption of the suprapatellar pouch [51, 70]. The mean QT autograft length found in the current study was 8.1 cm; this included all reported grafts whether harvested with or without a bone block. This is comparable to the mean lengths of 7.7 cm found in one study and 6.9 cm found in another [85, 88]. However, the latter study found that 33.7% of patients had inadequate QT length for a free QT autograft, while only 3.3% had inadequate length with a bone block [88]. Contrarily, while femoral tunnel length is not an exact measure of graft or tendon length, a recent systematic review showed that comparable post‐operative outcomes were achieved following ACLR with QT autografts between short and long femoral tunnel groups [8]. Moreover, our present study is in agreement with a recent systematic review reported that there are clinical outcomes or complication rates between full‐thickness and partial‐thickness QT autografts [41]. The impact of QT length on ACLR outcomes has not been fully uncovered and should be a focus of future research.

Our analysis demonstrates that MRI measurements are more strongly correlated with QT autograft parameters in comparison to anthropometric characteristics, yet neither seems to play a key role in predicting ACLR outcomes. All studies that reported on the relationship between MRI parameters and QT autograft parameters found a statistically significant relationship; most of these associations were found to have a moderate positive correlation. A minority of studies found a statistically significant relationship between anthropometric characteristics and QT autograft parameters; from these studies, all of the reported associations were of low positive correlation. In a similar study assessing the same correlations but with four‐strand hamstring autografts instead, it was found that 63.2% of studies reporting on MRI CSA demonstrated a moderate to very high positive correlation, whereas 25.7% of studies reporting on height found a moderate to very high correlation and 21.2% of studies reporting on weight reported a moderate correlation with graft diameter [82]. Our systematic review shows that on average, anthropometric characteristics are poor and MRI is variable in predicting QT autograft diameter. QT autograft parameters were also not found to be significantly associated with any negative post‐operative outcome or complication. Thus, based on these findings and the current literature, predicting intraoperative QT autograft parameters with either MRI or anthropometric characteristics may not be clinically important for predicting outcomes and complications following ACLR, and it may be worthwhile to shift our attention to other clinically relevant markers. However, not enough studies have investigated the effect of QT autograft size on post‐operative outcomes to justify this claim.

Recently some concern has been expressed surrounding the notion that QT autograft use may result in higher rates of post‐operative arthrofibrosis. However, there currently lacks a consensus within the literature and this relationship has yet to be fully elucidated. Findings from one study [33] show that QT autografts and QT autografts particularly with a femoral tunnel diameter of 9.25 mm or greater in male patients in another study [28] are statistically significantly associated with an increased risk of developing arthrofibrosis. Moreover, as females have a statistically smaller femoral notch when compared to males [14], care must be taken to avoid notch impingement [55]. Despite the above concerns, there is also evidence to the contrary; one study investigated this relationship and found no association between QT autograft size and arthrofibrosis [56]. This study does not substantiate the concern that larger QT autografts are associated with arthrofibrosis or other post‐operative complications; further investigation is needed to fully understand the impact of QT use on arthrofibrosis.

The present study was performed with an extensive systematic search of three medical databases and the use of independent authors to screen and extract data. Furthermore, there was an almost perfect agreement between the authors during the title and abstract stage and during the full‐text stage of screening. Additionally, this study is one of, if not the first systematic review to compare the association between MRI or anthropometric characteristics and QT parameters. Due to the emerging popularity of QT autografts, such data is vital to predict surgical outcomes in the future. However, while robust, the present study is not devoid of limitations. Most of the reviewed studies did not present data on QT autograft length which may be important for understanding a wider variety of predicted outcomes. Furthermore, of the studies which presented MRI data, a variety of techniques were used which may have skewed the extracted measurements. Finally, the majority of studies were of level III evidence, disallowing the pooling of data due to heterogeneity of methodologies.

## CONCLUSION

QT autografts used in ACLR routinely have a large mean diameter (>8 mm). Preoperative MRI measurements are better than anthropometric characteristics at predicting intraoperative QT autograft parameters, however this may not be clinically relevant. QT autograft parameters were not found to be associated with any post‐operative complication or outcome; further research is needed to evaluate the relationship between QT autograft use and arthrofibrosis, as well as between QT length and ACLR outcomes.

## AUTHOR CONTRIBUTIONS

**Joshua Dworsky‐Fried**: Screening; data extraction; writing; editing. **Amanda Hadwen**: Screening; data extraction; writing; editing. **Luca Bernardini**: Data extraction; writing; editing. **Prushoth Vivekanantha**: Writing, editing, idea conception. **Alberto Grassi**: Writing; editing. **Matthieu Ollivier**: Writing; editing. **Darren de SA**: Writing; editing; idea conception.

## FUNDING INFORMATION

There were no funding sources utilized in the development of this research.

## CONFLICT OF INTEREST STATEMENT

The authors declare no conflicts of interest.

## ETHICS STATEMENT

There are no relevant ethical disclosures pertaining to research involving human participants and/or animals, and informed consent was not necessary to develop this manuscript.

## Supporting information

Supporting information.anon

## Data Availability

Data may be made available upon reasonable request at prushoth.vivekanantha@medportal.ca.
