# Supplementary material for: Quadriceps tendon autograft diameters are routinely above 8 mm, and preoperative size estimation before anterior cruciate ligament reconstruction may not be necessary for this graft type: A systematic review
Source: Knee Surg Sports Traumatol Arthrosc. 2024 Dec 17;33(9):3111–33. doi: 10.1002/ksa.12558 (PMC12392381; doi:10.1002/ksa.12558)
Supplement: Supplementary file 1 — Supporting information.anon [file KSA-33-3111-s001.pdf]

SUPPLEMENTARY DIGITAL MATERIAL:

Supplementary Table 1. Search Criteria

**Search Criteria**
1. Quadricep
2. Anterior cruciate ligament OR ACL
3. Diameter OR length OR width OR size
4. #1 AND #2 AND #3

Supplementary Table 2. Statistical Associations Between Graft Parameters and Post-Operative Outcomes

**First Author (Year of publication)**	**Type of Statistics**	**Parameter Type**	**Outcome**	**Statistical Association**
Haley (2023)	Binomial logistic regression, ROC curve analysis	Continuous	Femoral tunnel size association with developing arthrofibrosis	Coefficient: 0.39<br>SE: 0.21<br>Wald Chi-square Test statistic: 3.41<br>Significance: 0.07<br>Odds ratio: 1.47<br>Lower CI: 0.98<br>Upper CI: 2.22
Lee (2024)	NR	NR	The thickness of the full-thickness QTPB graft was 8.0 ± 1.1 mm, which was thicker than the partial-thickness QTPB graft (5 mm). By multiplying the width of the graft (10 mm), the average cross-sectional area of the full-thickness and partial-thickness QTPB grafts was 80	NR

			mm2 and 50 mm2, respectively. The cross-sectional area of the partial-thickness QTPB graft (rectangular tunnel ACLR group) was 62.5% of the average cross-sectional area of the full-thickness QTPB graft (round tunnel ACLR group).	
Letter (2023)	Covariance analysis	Peak torque, average power, total work	Peak torque: FT-Q group, the operated side had significantly lower peak torque than the non operated side ($P < .001$), but no differences were seen between sides for the PT-Q group. significant side × depth interaction ($F[1,23] = 7.72$; $P = .011$; $\eta 2 = 0.251$). average power: significant interaction between side and depth ($F[1,23] = 7.33$; $P = .013$; $\eta 2 = 0.242$). significantly lower average power for the operated versus non operated side in the FT-Q group only ($P < .001$) total work: significant side × depth interaction ($F[1,23] = 7.31$; $P = .013$; $\eta 2 = 0.241$). Pairwise comparisons revealed that for the FT-Q group, the operated side had significantly less total work than the non operated side	p value < 0.05

			(P<0.001), but no significant difference was seen between sides for the PT-Q group.	
Pennock (2019)	Categorical variable	Graft failure	QT grafts failing less frequently than the hamstring tendon grafts (4% vs 21%, respectively; P = .037)	Graft diameter size was not significantly associated with ACL retears (P ¼ .12, p value = 0.37)
Pfeiffer (2023)	NR	NR	NR	Between the vancomycin concentration and the graft diameter (median, 8.5 mm; range, 6.0-10.0 mm; r = –0.026; P = .914 NS
Tang (2024)	Descriptive statistics, Mann-Whitney U test	Continuous	Graft size and flexor peak torque + extensor peak torque.	NR

Abbreviations: QTPB: Quadriceps Tendon-Patellar Bone, ACLR: Anterior Cruciate Ligament Reconstruction, QT: Quadriceps Tendon
